# Genetics and Genomics of Addiction

**DOI:** 10.3390/genes14091760

**Published:** 2023-09-04

**Authors:** Jason A. Bubier

**Affiliations:** The Jackson Laboratory, Bar Harbor, ME 04609, USA; jason.bubier@jax.org

Substance use disorders (SUD), like many neuropsychiatric conditions, are a heterogeneous group of disorders with similar symptomatology but often different pathoetiology. As diagnosed by the DSM-V, SUD requires at least two of eleven diagnostic criteria, resulting in the possibility of over 2000 different combinations of symptoms. Further segmenting the disorder is that SUD can involve a variety of legal and illegal substances. There are currently approaches to treating and managing SUD, but we have a limited armamentarium. Genetic and genomic studies of addiction can facilitate a more accurate molecular diagnosis and, ultimately, the development of appropriate treatments. Sequenced genomes, new animal model resources, and Genome Wide Association Studies (GWAS) of large sample sizes have made vast inroads into our understanding of the genetics that underlies addiction. Future mechanistic studies will be driven by integrating big data from animal models and human studies to identify their consilient features. This Special Issue, “Genetics and Genomics of Addiction,” focuses on genetic contributions to this disease that may lead to better-targeted therapeutics and diagnostics. This issue contains five original research articles and one review paper that furthers our collective knowledge of SUD disease etiology and the genetic risk factors underlying the disease.

Chunduri, Watson and Ashbrook explore how the controlled laboratory environments of animal model research can be leveraged to understand the gene-by-environment (GxE) interactions underlying SUD [1]. Their work relies on the recombinant inbred population of the BXD strains of mice, which have reproducible genomes that can be tested in different laboratory environments, allowing one to make novel insights into GxE. They illustrate that this can be accomplished using GeneNetwork.org, a database and tool suite containing over 10,000 phenotypes and over 100 ‘omics datasets measured under various environmental conditions derived from the BXD panel. The group reanalyzed data published in 2010 using new mapping software and complete genomic sequence and identified novel candidate genes involved in response to drug use. Also, using model organisms, Rizk et al. [2] explored the impact of sex and circadian disruption on alcohol drinking in mice. Utilizing both sexes of *Clock* mutants, the researchers assessed alcohol consumption and preference. As expected, female control inbred C57BL/6J mice drank more than the males. However, there were no sex differences in the *Clock* mutants, which displayed greater alcohol preference and consumption over controls of both sexes. Studies like these illustrate how the environment and circadian disruption can affect SUD. The final article in this collection utilizing animal models was the work of Lie et al. [3] in which the authors set out to determine the relationship between methylation of the guanine nucleotide-binding protein, α stimulating complex locus, (*Gnas)* promoter, *Gnas* mRNA levels and morphine reward memory. Hypermethylation of the *Gnas* promoter and decreased mRNA expression in the basolateral amygdala were necessary for reconsolidating opiate-associate memories. A review paper by Sambo and Goldman [4] summarizes the current knowledge around the role of genetic influence in Fetal Alcohol Spectrum Disorder (FASD). This SUD-adjacent field of study explores the range of outcomes that result from prenatal alcohol exposure. The human literature related to twin studies, copy number variations, and candidate gene studies, illustrates a role for various genetic factors in FASD susceptibility. Fedorenko et al. [5] also explored alcohol use, assessing the association of phosphatidylinositol-5-phosphate-4-kinase type two α (PIP4K2A) polymorphisms with alcohol use disorder. Polymorphisms in PIP4K2a, a gene known to play a role in dopaminergic pathways, were tested for their association with AUD in a population of Russian men. In this case-control study, two PIP4K2A SNPs had a higher association of occurring in cases than in controls, suggesting that different PIP4K2A polymorphisms may be associated with an increased risk of AUD. The final paper in this collection, that of Chmielowiec et al. [6], also performed case-control studies looking at the associations of polymorphisms in the dopamine transporter (DAT1) with new psychoactive substance use. New psychoactive substances are drugs designed to mimic established drugs, such as cannabis, cocaine, MDMA and LSD [7]. Their conclusion supported a role for certain DAT1 polymorphisms being associated with higher levels of neuroticism and new psychoactive substance use.

This special issue aimed to bring together researchers of substance use disorder across various drugs of abuse and experimental systems to describe the current status of addiction genetics. Psychiatric disorders are known to be complex traits due to their polygenic nature. The articles in this special issue covered various topics and provided diverse insights to direct future SUD genetics research. The growing number of pathways, genes, proteins, and molecules that appear to be involved in SUD is of special interest. We anticipate that this Special Issue will help researchers search for additional genetic associations that will help refine our understanding of the etiology of this complex disease.
